# Changes to lung surfactant monolayers upon exposure to gas phase ozone observed using X-ray and neutron reflectivity

**DOI:** 10.1039/d2ea00032f

**Published:** 2022-06-14

**Authors:** Joanna M. Hemming, Justyna Szyroka, Gracia Shokano, Thomas Arnold, Maximilian W. A. Skoda, Adrian R. Rennie, Katherine C. Thompson

**Affiliations:** Institute of Structural and Molecular Biology, Department of Biological Sciences, Birkbeck, University of London Malet Street London WC1E 7HX UK k.thompson@bbk.ac.uk; European Spallation Source The ESS Campus Lund Sweden SE-221 00; ISIS Neutron and Muon Source, Science and Technology Facilities Council, Rutherford Appleton Laboratory Harwell, Didcot Oxford OX11 0QX UK; Department of Chemistry, University of Bath Claverton Down Bath Avon BA2 7AY UK; Department of Chemistry – Ångström and Centre for Neutron Scattering, Uppsala University Box 538 75121 Uppsala Sweden

## Abstract

Exposure to the secondary pollutant ozone in ambient air is associated with adverse health effects when inhaled. In this work we use surface pressure measurements, combined with X-ray and neutron reflection, to observe changes in a layer of lung surfactant at the air water interface when exposed to gas phase ozone. The results demonstrate that the layer reacts with ozone changing its physical characteristics. A slight loss of material, a significant thinning of the layer and increased hydration of the surfactant material is observed. The results support the hypothesis that unsaturated lipids present in lung surfactant are still susceptible to rapid reaction with ozone and the reaction changes the properties of the interfacial layer.

## Abstract 2

Lung surfactant at the air water interface reacts with ozone leading to changes in surface tension, film thickness, structure.

## Graphical abstract

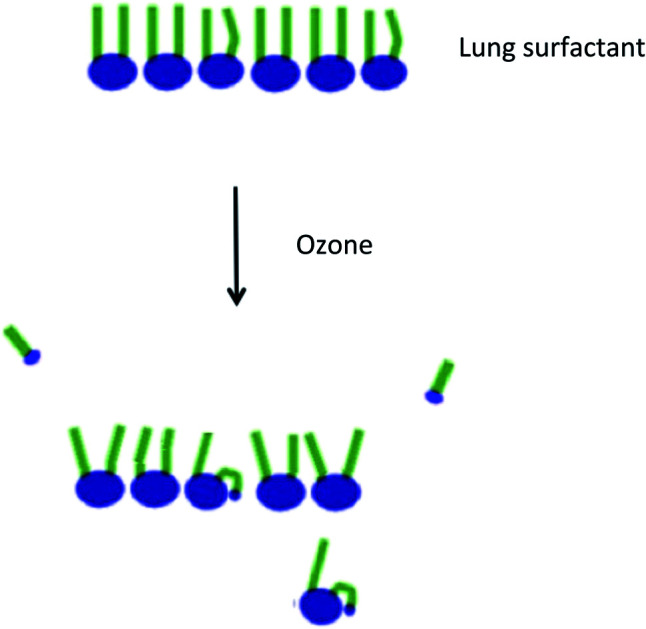

**Environmental significance**

In our article we show how X-ray and neutron reflection can be used to follow changes to lung surfactant as it is exposed to the ubiquitous environmental pollutant ozone. The presence of ozone in ambient air, and the detrimental impact it has on our health, is a key environmental concern. Our manuscript reveals how inhaled ozone could interact with, and damage, the layer of lung surfactant lying at the outside surface of the lung.

## Introduction

The alveoli of the lungs provide the large surface area to volume ratio required to enable sufficient transfer of oxygen into the blood stream and carbon dioxide out. The outer surface of the alveoli are coated in a fluid called lung surfactant, which contains a mixture of lipids and proteins. A monolayer of lipids and hydrophobic proteins from this surfactant lies at the interface between the external air and the aqueous subphase, acting as the first barrier which must be crossed by the inhaled gases. The lipids and proteins in the monolayer lower the surface tension of the interface when the lungs are compressed; this reduces the effort required to expand and contract the lungs and prevents alveoli collapse, and the respiratory distress this would cause.^1^ A wide variety of lipids and several different proteins are found in lung surfactant and the interaction between the components is key to its function.^2^ The main species present at the air–water interface are the saturated lipid 1,2-dipalmitoyl-*sn*-glycero-3-phosphocholine, DPPC, a range of unsaturated phospholipids such as 1-palmitoyl-2-oleoyl-*sn*-glycero-3-phosphocholine, POPC, and 1-palmitoyl-2-oleoyl-*sn*-glycero-3-phospho-(1′-rac-glycerol), POPG, cholesterol and two surfactant proteins, surfactant protein B, SP-B, and surfactant protein C, SP-C.^3^ Inhaled air must first cross this interfacial layer of lung surfactant before reaching the blood stream and any reactive species present in the air may react with the surfactant layer potentially damaging it.

Ozone is present in all outdoor ambient air but the concentration can be very much elevated when pollutants are present. Ozone itself is not generally emitted directly into the air as a primary pollutant, although it can be in some cases, rather ozone is a secondary pollutant found in photochemical smog, where it is formed from the photolysis of NO_2_:

1 NO_2_(g) + sunlight → NO(g) + O

2 O + O_2_ → O_3_

The NO formed in this reaction can be converted back into NO_2_ through reaction with alkoxy radicals formed during the oxidation of volatile organic compounds in the atmosphere, thus a cyclic system emerges where O_3_ is catalytically formed when oxides of nitrogen and volatile organic compounds are present in ambient air on sunny days. Sources of nitrogen oxides in the atmosphere include the burning of petrol (gasoline) in internal combustion engines, which can also be a source of volatile organic compounds, although vegetation is the dominant source of these. Fuller descriptions of the processes that lead to high levels of ozone in ambient air can be found in one of the many review articles that have been written on the subject.^4^ The World Health Organization, WHO, states that the 8 hours daily maximum for ozone in outdoor air should not exceed 100 μg m^−3^ (∼50 ppb).^5^ Levels of ozone in ambient air vary greatly depending on location, season, time of day and local weather, however hazardous levels are encountered frequently across the world. Measures to control ozone have been reasonably effective in the past, with levels in Mexico city now peaking in the 100 ppb range when back in early 1990s levels approaching 500 ppb were frequently encountered.^6^ However ozone is still being formed in significant levels in ambient air and background levels in the UK are currently rising gradually over the last few years, as indeed they are in China and levels in excess of WHO limits are encountered in regions across the globe.^7,8^

The link between ozone in ambient air and adverse health effects has been well documented, for instance Bell *et al.*, in 2004 and Turner *et al.*, in 2016 have both analysed very large data sets and found a statistically significant association between ozone levels and mortality from respiratory causes.^9,10^ Recent studies indicate that long-term exposure to even low levels of ozone leads to a decrease in lung function in children and that limiting levels in ambient air to 70 ppb may not be adequate to prevent this.^11^ The first barrier that the inhaled ozone reaches is the layer of lung surfactant at the air/aqueous interface. A few review articles summarise what is currently known about the interaction of ozone with lung surfactant.^12,13^ Only a relatively small number of studies have focused on changes to lung surfactant, and model lipid and lipid protein mixtures, at the air/aqueous interface itself when exposed to ozone.^14–22^ Changes in the surface pressure, *π*, were recorded when lipid monolayers, or in one case a lipid/peptide monolayer, at the air/aqueous interface were exposed to low levels of ozone gas.^14,15,17,20,21^ The surface pressure is related to the surface tension, *γ*, by *π* = *γ*_0_ − *γ*, where *γ*_0_ is the surface tension of the clean aqueous solution. A few groups have used mass spectrometry, either indirectly,^14,15^ or directly,^18,19^ or surface specific spectroscopy techniques^22^ to follow the product formation. In just a few cases have either X-ray or neutron reflection been used^16,17,20,21^ and there are no studies that we are aware of where changes to the physical properties of natural lung surfactant have been directly observed using X-ray or neutron reflection during exposure to ozone gas, as we do here. In this work we deposited natural lung surfactant, recovered from animal lungs, at the air water interface and monitored changes to the surface pressure and the X-ray and neutron reflectivity of the layer as it is exposed to gas phase ozone.

## Materials and methods

**Fig. 1:**
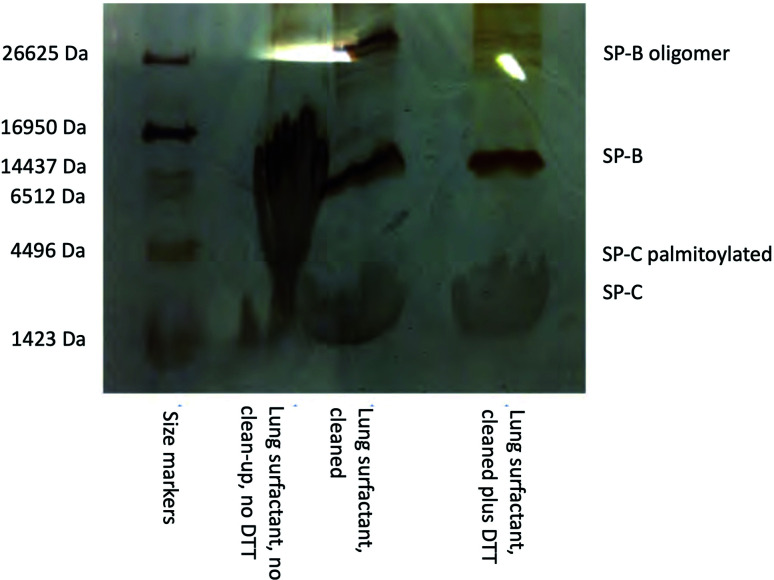
SDS-PAGE gel with tricine buffer system and silver staining showing the presence of the lung surfactant proteins SP-B and SP-C in extracted porcine lung surfactant. The use of the SDS-PAGE clean-up kit to remove lipids, and the reductant DTT (dithiothreitol), before electrophoresis greatly improved the appearance of the bands, the process also removes the palmitoyl groups from the SP-C protein.

The water used to prepare the buffer solutions had a resistivity of 18 MΩ cm and the solvents used were HPLC grade or higher. Fresh animal lungs were purchased from a butcher. The lung surfactant material was recovered by lavaging the bronchioles of the lungs with a saline solution and then extracting the lipids and hydrophobic SP-B and SP-C protein components according to the protocol of Bligh and Dryer.^23^ Briefly, the recovered saline mixtures were centrifuged at low speed (∼78×*g*) for 10 minutes to remove any large debris and the supernatant was recovered. The following reagents were then added in sequence, and the solution mixed well using a vortex between each addition: 3.75 volumes of 1 : 2 v/v chloroform : methanol solution, 1.25 volumes chloroform, 1.25 volumes water. The resulting mixture was centrifuged at ∼78×*g* for 5 min and the lower organic phase containing the surfactant lipids and proteins was recovered. An aliquot was reserved to verify the presence of SP-B and SP-C by SDS-PAGE using the tricine buffer system with silver staining^24^ and the remainder stored in a −20 °C freezer until required. A typical gel image for porcine lung surfactant showing the presence of bands corresponding to SP-B and SP-C is shown below in Fig. 1. Cleaning up the SDS-PAGE samples to remove associated lipids using an SDS-PAGE clean-up kit (GE Healthcare) greatly improved the band appearance.

Monolayers of the extracted lung surfactant were formed on 50 mM phosphate buffered pH 7 water at the air–water interface contained in a Langmuir trough. The initial solutions of surfactant were concentrated by evaporating off roughly 90% of the chloroform solvent under a stream of dry nitrogen. A few tens of microliters, depending on the trough size, were then spread on the aqueous subphase dropwise using a glass Hamilton syringe. The remaining solvent was allowed to evaporate. The surface pressure was recorded using a microbalance and a Wilhelmy plate composed of Whatman filter paper. The barriers of the Langmuir trough could be adjusted to vary the surface area available to the surfactant monolayer, and hence the initial surface pressure. A range of initial surface pressures were used, all below 35 mN m^−1^. In the experiments reported here the barriers were kept fixed during an experiment, hence the area of the monolayer was fixed as the surfactant was exposed to ozone. The Langmuir trough was housed in a box with inlets and outlets for gas. The ozone was generated by passing a 1 L min^−1^ stream of molecular oxygen past a UV lamp (UVP), thus generating small amounts of O_3_ in the O_2_ stream that were flowed into the box housing the trough. The exhaust gas from the box flowed into an extraction system. The surface pressure and neutron or X-ray reflectivity were recorded as the surfactant layer was exposed to the ozone gas in oxygen. Control experiments were performed where the surfactant was exposed just to oxygen. All experiments were performed at 21 ± 2 °C. To follow changes in the thickness and composition of the monolayer the reflectivity of the layer to both neutrons and X-rays was recorded, techniques which are well documented elsewhere.^25^ Reflection will occur when there is an interface between materials with different refractive index, a property that depends on the wavelength of the X-rays or neutrons and the scattering length density of the material. In the case of a thin layer on an aqueous subphase the reflectivity depends not just on the scattering length density of the various materials but also on the thickness of the interfacial layer. In the case of X-rays the scattering length density depends on the electron density of the material and so X-rays will be reflected off a clean air/aqueous surface and one must compare the signal obtained when X-rays are reflected from a layer of surfactant at the air/aqueous solution interface to that obtained for the clean surface. The scattering length density of a material to neutrons however depends on the neutron scattering length, *b*, which varies not just between atoms, but between isotopes. A useful feature is that the scattering length of ^1^H is of opposite sign to ^2^H, and so by mixing the appropriate proportions of H_2_O and D_2_O it is possible to create a subphase with the same refractive index as air, termed null reflecting water. The benefit of this is that any reflection observed can be directly attributed to the material spread at the interface. Neutron reflectivity experiments were performed at the ISIS Pulsed Neutron and Muon Source, Oxfordshire, UK on the INTER reflectometer^26^ whilst X-ray reflectivity measurements were performed on the I07 reflectometer^27,28^ at the Diamond Light Source, Oxfordshire, UK. In the neutron reflection experiments data were collected for a range of neutron wavelengths between 1 and 16 Å, and at two incident angles, *θ*, of 0.8° and 2.3°. For the X-ray reflection experiments data were collected for ∼1 Å (12.5 keV) but over a range of incident angles, thus in both cases the reflectivity of the material was obtained as a function of momentum transfer *q*, where *q* = (4π/*λ*)sin *θ*. The surfactant layer was damaged directly by prolonged exposure to the synchrotron X-ray beam and so the beam was rastered over the surface during an experiment.

X-ray reflectivity data were analyzed using the Motofit package,^29^ whilst neutron reflectivity data were analyzed using both the mono code,^30^ and the Motofit package, both of which use an optical matrix formalism to fit layer models of uniform layers representing the interfacial structure. In this approach the interface is described as a series of slabs, each of which is characterized by its scattering length density (SLD), *ρ*, thickness, *τ*, and roughness. A least-squares minimization is used to adjust the fit parameters to reduce the differences between the model reflectivity and the data. The fitted thickness and SLD values can be converted into surface concentration (*Γ*) values using the following relationship:^31^3
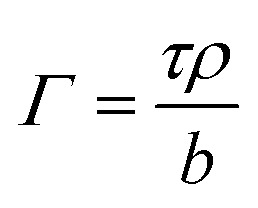


The program mono can fit directly the scattering length, *b*, per unit area and thickness that as explained by Crowley^32^ represents the optimised set of uncorrelated parameters. Even if the scattering length, *b*, of the molecules in the interfacial layer is not known, the relative amount of material given by the scattering length per unit area as it varies with time can be evaluated provided the chemical composition is substantially unchanged.

## Results and discussion

**Fig. 2:**
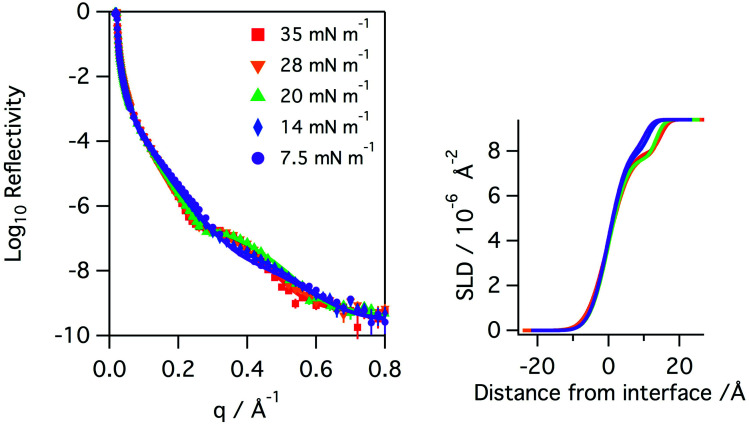
Left: X-ray reflectivity curves recorded for a monolayer of porcine lung surfactant on aqueous buffer at surface pressures of 35 (red squares), 28 (orange triangles down), 20 (green triangles up), 14 (blue diamonds) and 7.5 (purple circles) mN m^−1^. The measured data points are shown as solid circles and the lines represent the fits to the data. Right: profiles of the scattering length density, SLD, as a function of distance from the interface.

Monolayers of natural porcine and ovine lung surfactant, at low to medium surface pressures, have been studied using both X-ray and neutron reflection whilst being exposed to gas phase ozone. The monolayers of lung surfactant spread at the air–water interface gave rise to the expected increase in surface pressure as the area of the monolayer (and thus area per molecule) was reduced. The change in the shape of the X-ray reflectivity curves as the layer is compressed is seen in Fig. 2. Fitting of the recorded X-ray reflectivity data as a single layer of material on water, with roughness at both the subphase/layer and layer/air boundaries revealed that the layer increased in thickness steadily from 10.4 ± 2.0 Å to 14.7 ± 2.7 Å as the pressure was increased from 7.5 to 35 mN m^−1^. The scattering length density changed only slightly, decreasing from 8.25 ± 0.54 × 10^−6^ at 7.5 mN m^−1^ to 7.99 ± 0.47 × 10^−6^ Å^−2^ at 35 mN m^−1^. The roughness in the model of the layer was fixed at 4.2 Å for surface pressures of 7.5 to 20 mN m^−1^, but was increased to 4.8 Å for the higher surface pressures. The profiles for scattering length density as a function of distance from the interface are shown in the right panel of Fig. 2.

**Fig. 3:**
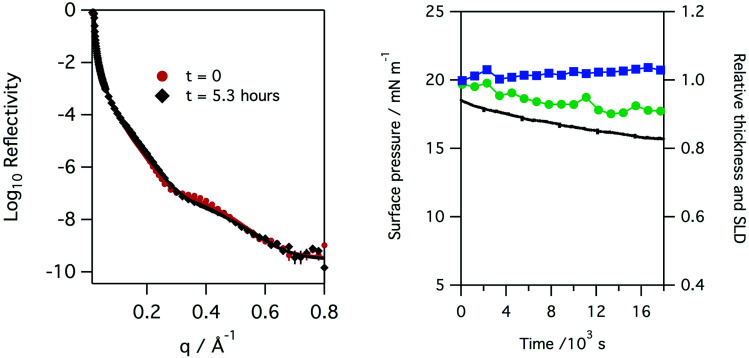
Left: X-ray reflectivity measured for a monolayer of porcine lung surfactant at the air/water interface at the start and after 5.3 hours of exposure to oxygen gas in an environment where the gas is exposed to the X-ray beam and hence low levels of oxidants can be formed. A slight change in the reflectivity is observed over this time period. Right: plot of surface pressure (black line), fitted relative thickness (green circles) and scattering length density, SLD, (blue squares) with time for the monolayer, a slight decrease in monolayer thickness and very slight increase in scattering length density is observed.

The neutron reflectivity of monolayers of natural lung surfactant at the air–liquid interface, held at constant area and exposed to a flow of pure oxygen showed little change over the course of several hours. However, exposure of the oxygen gas to the X-ray beam, as occurred during X-ray reflectivity measurements, is expected to lead to the generation of low levels of ozone. The monolayers of surfactant in the X-ray reflectivity experiments showed a gradual reduction in the surface pressure and thickness of the surfactant layer over the course of several hours, as shown in Fig. 3. Note, the X-ray beam itself probed a slightly different position of the surfactant surface each time a reflectivity curve was measured, as mentioned previously, so a change in reflectivity is not attributed to direct damage of the monolayer by the X-rays, but rather the damage caused by any gaseous oxidants formed by the action of the beam with molecular oxygen which once formed would be free to diffuse to and react with the surfactant layer.

**Fig. 4:**
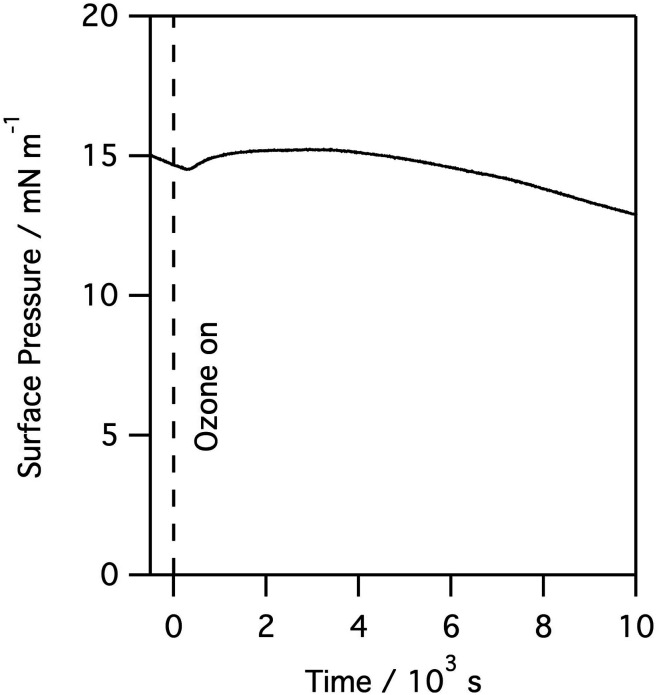
Surface pressure measured for a monolayer of porcine lung surfactant at the air/water interface when exposed to 100 ppb of gas phase ozone.

When monolayers of natural lung surfactant spread on aqueous buffers were deliberately exposed to higher levels of ozone gas a clear and rapid reaction was observed. Exposure leads to a rapid and continuous change in the surface pressure data. The exact changes in surface pressure varied between porcine and ovine surfactants, presumably reflecting the different lipid compositions. However, for all surfactants the largest and most noticeable feature observed was a pronounced rise in surface pressure followed by a much slower and steady decline, and this was seen even at very low ozone levels, *e.g.* 100 ppb, as seen in Fig. 4. The rate and extent of the increase in the surface pressure correlated with the ozone concentration and it was consistently observed for all levels of ozone, and all surfactant layers tested, including both porcine and ovine lung surfactant.

**Fig. 5:**
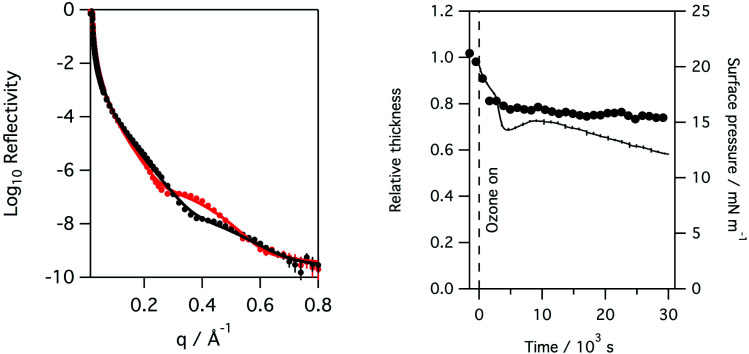
Left: reflectivity of a monolayer of porcine lung surfactant at the air/water interface to X-rays at the start and after 8 hours of exposure to 0.550 ppm of ozone in oxygen gas. A significant change in the reflectivity is observed over this time period. Right: plot of surface pressure (black line), fitted relative thickness (black circles) with time for the monolayer, a rapid drop to about 80% of the initial thickness is observed almost immediately upon exposure to the additional ozone after which the monolayer appears to remain at constant thickness.

**Fig. 6:**
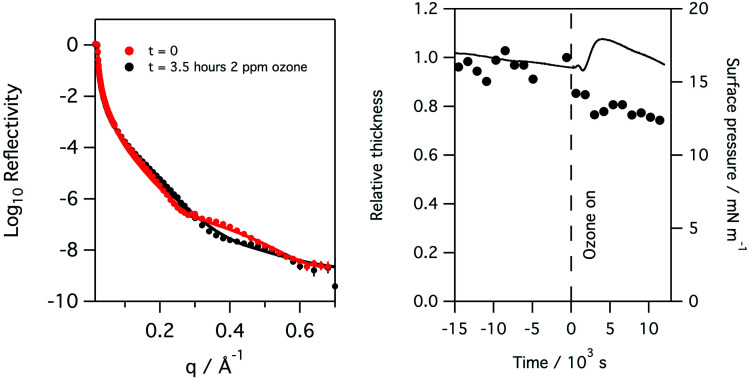
Left: reflectivity of a monolayer of ovine lung surfactant at the air/water interface to X-rays at the start and after 3.5 hours of exposure to 2 ppm of ozone in oxygen gas. Right: plot of surface pressure (black line), fitted relative thickness (black circles) with time for the monolayer. As in the case of the porcine surfactant, a rapid drop in layer thickness is observed upon exposure to ozone.

Recording the X-ray reflectivity of a lung surfactant monolayer when deliberately exposed to ozone gas, in addition to any formed by the action of the X-ray beam, showed that the exposure led to significant thinning of the surfactant layer to 80%. This is shown in Fig. 5 when the monolayer is exposed to 0.550 ppm of ozone over 8 hours. The thinning of the layer however happens very rapidly. Fig. 6 shows the same rapid drop in thickness occurs when a monolayer of ovine lung surfactant is exposed to ozone.

**Fig. 7:**
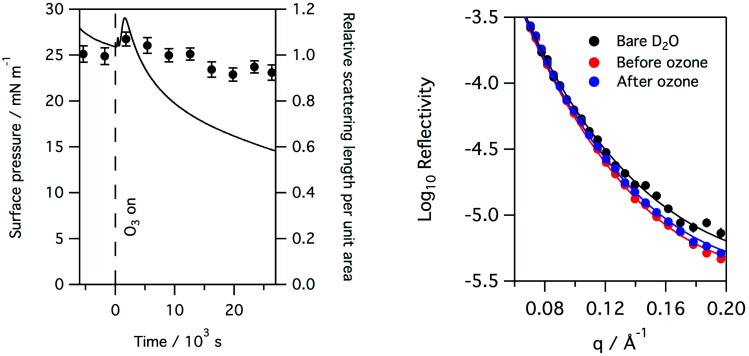
Left: the surface pressure and relative amount of material, as determined by neutron reflectivity, of a monolayer of ovine lung surfactant spread at the air–aqueous interface on null reflecting buffered water, as it is exposed to 1.1 ppm ozone. The surface pressure is shown as a solid black line and shows the increase in surface pressure on exposure to ozone followed by a slow decline. The scattering length per unit area, divided by the initial scattering length per unit area, obtained by fitting the reflectivity data, is shown by filled black circles and gives a measure of the amount of material at the interface as detectable by neutron reflectivity, see main text for further details. The initial slight increase in material shown is most likely caused by the loss of CH_2_ groups which have a negative scattering length density. Right: the reflectivity to neutrons of a clean buffered D_2_O surface, the surface with a layer of lung surfactant and the layer after exposure to 1.1 ppm ozone gas for 8 hours. The reflectivity of the surfactant layer increases slightly upon exposure indicating increased hydration of the surfactant layer by D_2_O.

The X-ray reflectivity measurements, which give useful data out to large values of *q* (∼0.8 Å^−1^), were used to follow changes in the thickness of the surfactant layer. The significantly higher background, and lower beam intensities, in neutron reflection experiments limit the useful range of *q* to about 0.3 Å^−1^, at least in the instrument set-up employed in this work, and so thickness is not as precisely determined using neutron reflectivity for the thin films considered here. However, the availability of a null reflecting subphase for neutrons, as described above, is a major benefit of the technique as the reflection is only from the material at the interface, and the product of the thickness and scattering length density for the interfacial material can be related to the amount of material at the interface. In this work natural lung surfactants were used, thus the interfacial material was not deuterated and the reflected signal was low. Taking the lipid DPPC as a typical component of lung surfactant, the head group has a scattering length density of ∼1.8 × 10^−6^ Å^−2^, but the tails have a scattering length density of −3.6 × 10^−7^ Å^−2^, leaving the overall scattering length density for the lipid to be just 2.4 × 10^−7^ Å^−2^. Fig. 7 below shows the change in the product of the scattering length density and thickness, a proxy for the amount of material at the interface as observed by neutron reflectivity, this has been obtained by fitting the reflectivity curves when a monolayer of ovine lung surfactant is exposed to 1.1 ppm gas phase ozone. A slight increase in material, as detectable by neutrons, is observed initially followed by a very slow decay. This increase is attributed to the increase in scattering length density of the surfactant layer caused by the loss of CH_2_ groups following reaction with ozone, as the scattering length density of a CH_2_ group is negative. The right panel of Fig. 7 shows the reflectivity of a clean buffered D_2_O surface, the surface with a layer of lung surfactant deposited and the layer after exposure to 1.1 ppm of ozone for 8 hours. Exposure to ozone has led to a slight increase in the reflectivity, *i.e.* the layer becomes more similar to D_2_O after exposure to the ozone, indicating increase hydration of the surfactant upon exposure to ozone.

**Fig. 8:**
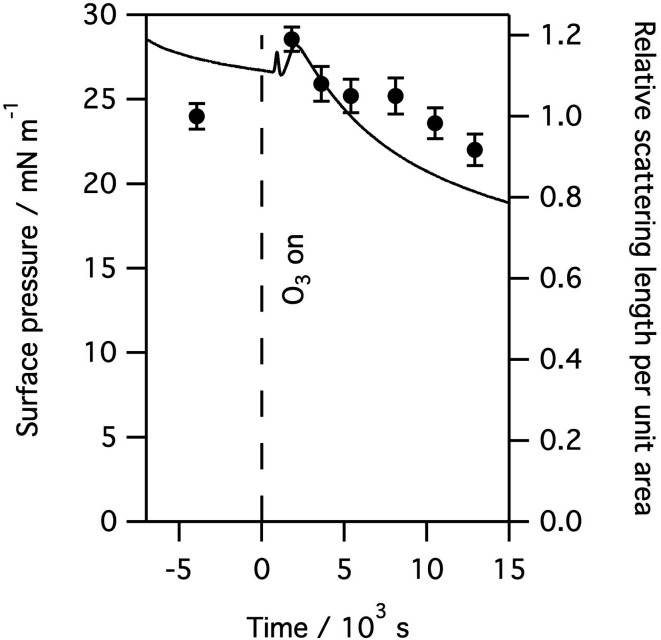
Surface pressure and relative material, as determined by neutron reflectivity, of a monolayer of porcine lung surfactant spread at the air–aqueous interface on null reflecting buffered water, as it is exposed to 1.1 ppm ozone. The surface pressure is shown as a solid black line and shows the increase in surface pressure on exposure to ozone followed by a slow decline. The scattering length per unit area divided by the initial value, obtained by fitting the reflectivity data, is shown by filled black circles and gives a measure of the amount of material at the interface as detectable by neutron reflectivity, see main text for further details.

As can be seen from Fig. 7, exposure to ozone leads to a rapid and dramatic change in the surface pressure and a matching change in the amount of material at the interface, that is both show initial increases and then exhibit a slow decline. Although the example above is for a layer of ovine surfactant, very similar profiles for the amount of material at the interface were obtained for porcine surfactant samples, for instance see Fig. 8. The change in the surface pressure showed a little variation with sample, although the dominant change was the same in all cases.

Considering together the results obtained from the three techniques: surface pressure measurement, X-ray reflectivity and neutron reflectivity allows us to reach some more insightful conclusions of the changes that occur to lung surfactant at the air–water interface when exposed to ozone gas. First, clearly evident from the changes in surface pressure that were observed for all lung surfactant samples studied and at all levels of ozone down to 80 ppb, well within the range encountered in polluted ambient air, is that the lung surfactant reacts with ozone and a change in the interfacial layer occurs. The dominant feature observed was an initial rise in surface pressure followed by a slow decline, exactly as observed when monolayers of the unsaturated phospholipids POPC and POPG, both of which are present in lung surfactant, are exposed to ozone at the air water interface.^17,20,21^ It seems logical therefore to attribute this change in surface pressure to the rapid reaction of ozone with the unsaturated phospholipid component of the natural lung surfactant. It has been shown by others that the presence of unsaturated lipids in lung surfactant is essential and thus loss of these lipids from the interface is of concern, even if they will be replenished.^2^ The other, more minor, changes observed in this work to the surface pressure when surfactant layers are exposed to ozone may be attributed to the reaction of ozone with other components in the lung surfactant. We have previously shown that the presence of lung surfactant protein B, SP-B, at the air–water interface leads to a slight drop in surface pressure when exposed to ozone.^21^

The reaction of ozone with unsaturated phospholipids at the air–water interface leads to a range of products. Early work showed that reaction with ozone led to cleavage at the site of the double bond and recovery and analysis of the material remaining at the interface by mass spectrometry found it to contain an aldehyde or carboxylic acid functional group terminating the lipid tail at the position previously held by the double bond.^14^ Later, *in situ* spectroscopic work revealed that it is the aldehyde group that is formed from the direct reaction of ozone with POPC,^22^ and *in situ* mass spectroscopic analysis also reveal hydroxyhydroperoxides and secondary ozonides are initially formed.^18^ The terminal portion of the tail (beyond the original double bond) has previously been shown by us, using isotopic labelling and neutron reflection, to leave the interface.^20^ As the alkyl tails of lipids contribute in a negative way to the overall scattering length density of the lipid, loss of a portion of the lipid tail would lead to an increase in scattering length density of the interfacial material and thus an apparent increase in material at the interface as determined by neutron reflection, which is exactly what is initially observed, see Fig. 7 and 8. The loss of the terminal portion of the lipid tails, as the area per molecule was fixed during the experiments, led to a slight collapse of the lipid tails and thus results in a thinning of the film, again exactly as we observed in the X-ray reflectivity profiles, Fig. 5 and 6. The X-ray reflectivity, and neutron reflectivity experiments on D_2_O subphases, also indicate that exposure of the lung surfactant layers to ozone lead to a slight increase in the hydration of the surfactant layer. In summary, the reaction leads to damage to the surfactant as seen by changes in the surface pressure, thinning of the surfactant layer and increased hydration of the head regions. These results are consistent with the ozone reacting with unsaturated phospholipids present in the surfactant, leading to cleavage of the double bonds and loss of the terminal portion of the lipid tail from the interface. The more complicated details of the profiles observed for the surface pressure for different surfactants indicate that in addition to reaction with the unsaturated phospholipids other reactions also occur in the monolayer, possibly the reaction of the lung surfactant proteins and other lipids such as cholesterol with ozone, which is a focus for future work. *In vivo* the situation is more complicated still as below the monolayer of surfactant material lies a reservoir of lipids and proteins, that will be associated with the interfacial layer and respond to it. This is discussed in some detail in the review by Cañadas *et al.*^2^ In addition to interactions of the damaged interfacial layer with the reservoirs, and possible replenishment of the lipids at the interface, there is also the possibility that some inhaled ozone will not react with the interfacial layer, but instead manage to cross over into the subphase, where it could potentially react with the materials there.

## Conclusions

Monolayers of natural lung surfactant spread at the air/aqueous solution interface have been studied using surface pressure measurements combined with X-ray and neutron reflection measurements. The results indicate that exposure to ozone, even at levels found in ambient air, leads to reaction of the surfactant in the interfacial layer. A change in surface pressure occurs, along with thinning and increased hydration of the surfactant layer. The results are consistent with reaction of ozone with the unsaturated phospholipids in the surfactant, which we have previously shown leads to cleavage of the lipid tails at the site of the double bond, followed by loss of the terminal portion of the tail from the interface, and thus to a rearrangement of the remaining lipid at the interface. The results demonstrate that natural lung surfactant does indeed react as predicted by the model surfactant systems previously studied.

## Conflicts of interest

There are no conflicts to declare.

## Supplementary Material
